# Progression and metastasis of lung cancer

**DOI:** 10.1007/s10555-016-9618-0

**Published:** 2016-03-28

**Authors:** Helmut H. Popper

**Affiliations:** Research Unit Molecular Lung and Pleura Pathology, Institute of Pathology, Medical University of Graz, Auenbruggerplatz 25, Graz, 8036 Austria

**Keywords:** Lung cancer, Angiogenesis, Metastasis, Hypoxia, Migration, Circulation, Epithelial-mesenchymal transition, Brain, Bone

## Abstract

Metastasis in lung cancer is a multifaceted process. In this review, we will dissect the process in several isolated steps such as angiogenesis, hypoxia, circulation, and establishment of a metastatic focus. In reality, several of these processes overlap and occur even simultaneously, but such a presentation would be unreadable. Metastasis requires cell migration toward higher oxygen tension, which is based on changing the structure of the cell (epithelial-mesenchymal transition), orientation within the stroma and stroma interaction, and communication with the immune system to avoid attack. Once in the blood stream, cells have to survive trapping by the coagulation system, to survive shear stress in small blood vessels, and to find the right location for extravasation. Once outside in the metastatic locus, tumor cells have to learn the communication with the “foreign” stroma cells to establish vascular supply and again express molecules, which induce immune tolerance.

Lung carcinomas when detected are most often in a metastatic stage IV. Lung carcinomas metastasize by lymphatic as well as blood vessels. When careful evaluation is done in resected lung carcinomas, vascular invasion is often seen in low-stage tumors, which usually results in increased incidence of recurrence as well as shortened survival of the patient [1]. Whereas metastasis *via* the lymphatic route usually takes longer until distant metastases are set, spreading *via* blood vessels will set early on distant metastases. Lung carcinomas have some preferential sites for metastasis, such as the brain, bones, and adrenal glands. Other organs are involved usually in late stage of the disease. Within the different types of lung carcinomas, there is also a preferential metastatic site, such as liver metastasis in small-cell lung carcinoma (SCLC) and brain metastasis in SCLC and adenocarcinoma [2–4]. In recent years, brain metastasis are increasingly seen in adenocarcinomas with epidermal growth factor receptor (EGFR) mutations and EML4ALK1 rearrangement, whereas squamous cell carcinomas in many cases have a tendency to locally invade the thoracic wall [4, 5]. This opens a variety of questions on metastasis in lung carcinomas, which we aim to address in this review.

When dissecting metastasis into developmental steps, there are several ways to approach this theme, including the first step of invasion into the stroma. Due to space limits, we will not discuss the process of precursor to *in situ* carcinoma transition and also will not focus on stroma invasion. We will focus on

- Tumor establishment and cell migration, followed by
- Vascular invasion—lymphatic and hematologic,
- Extravasation, and finally, end with
- Creating the distant metastatic focus.

## Tumor establishment and cell migration

After tumor cells have invaded the stroma, several tasks have to be organized. To promote tumor growth, the tumor cells need to organize vascular supply for nutrition and oxygen uptake. For movement within the stroma, this needs to be restructured; the tumor cells have to escape lymphocytic attacks; and finally, for migration, the tumor cells have to adapt to a migratory cell structure.

### Angiogenesis, hypoxia, and stroma (microenvironment)

**Fig. 1:**
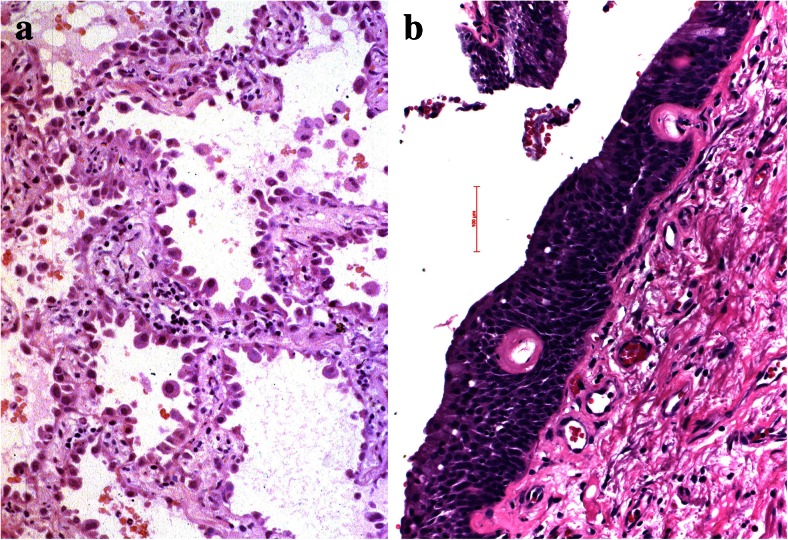
Angiogenesis in preneoplastic lesions, **a** atypical adenomatous hyperplasia has no new vessels but instead relies on the normal vascular architecture of preexisting alveolar septa; in the vascular variant of squamous cell dysplasia, **b** the preneoplastic cells induce angiogenesis using vascular growth factors produced by the dysplastic cells

**Fig. 2:**
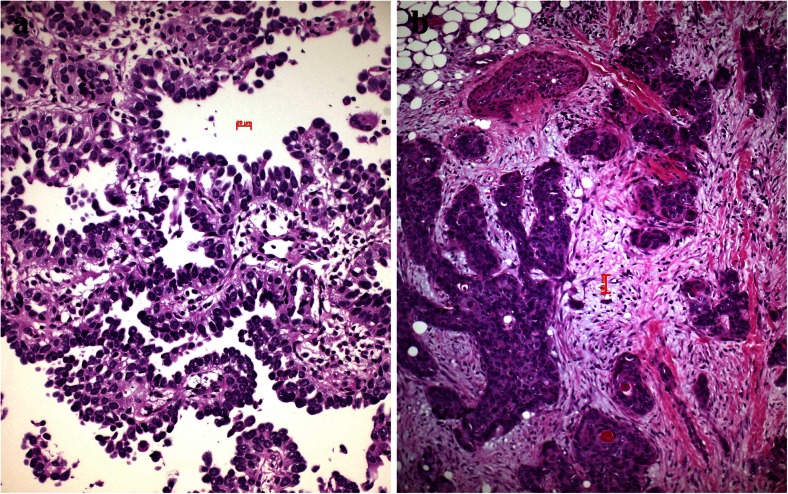
Desmoplastic stroma reaction is almost absent in this well-differentiated lepidic predominant adenocarcinoma (**a**) whereas prominent in this squamous cell carcinoma (**b**)

When tumor cells start to form nodules within the stroma, they need to communicate with the surrounding microenvironment, which is composed mainly by macrophages, fibroblasts/myofibroblasts, neutrophils, lymphocytes, and dendritic cells. To facilitate angiogenesis, tumor cells can either directly release angiogenic factors such as vascular endothelial growth factors (VEGFs) to directly stimulate the formation of new blood vessels, or tumor cells cooperate with macrophages, which can release angiogenic growth factors [6–8]. A good example for angiogenesis induced by tumor cells is the vascular variant of squamous cell dysplasia, whereas in well-differentiated adenocarcinomas, angiogenesis seems to relay on cooperating macrophages [9–12] (Figs. 1a, b and 2a). To understand the function of macrophages, it is necessary to briefly discuss the two different populations of macrophages, the M1 and M2 types. M1 macrophages are acting against tumor cell invasion by secreting interleukin 12 (IL-12), which function tumoricidal by an interaction with cytotoxic lymphocytes and NK cells. M2 macrophages produce IL-10, which promote tumor progression. The differentiation of naïve macrophages into either M1 or M2 types is facilitated by NOTCH, where low Notch *via* SOCS3 drives macrophages into M2 types [13]. M1 macrophages act proinflammatory, inactivate autophagy by production of radical oxygen species, and can also induce apoptosis of tumor cells [14–16]. Notably, mutation and inactivation of Notch are found in neuroendocrine carcinomas, whereas activation in other non-small-cell carcinomas, which questions the function of this gene as either oncogene or tumor suppressor [17–20]. Most probably different members of the Notch family proteins function differently in squamous cell, small cell, and adenocarcinomas and in addition, act differently during tumor development [21–23].

#### The role of hypoxia in tumor cell migration and metastasis

**Fig. 3:**
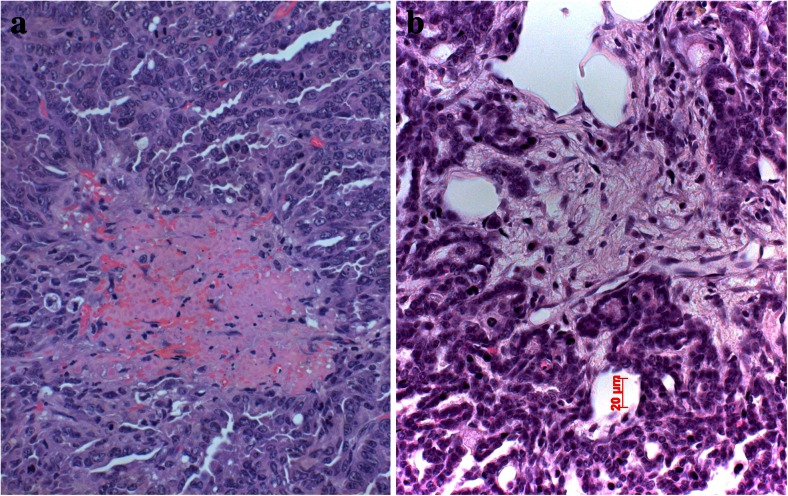
Experimental adenocarcinoma in a mouse. Carcinoma is induced by mutant KRAS. At a certain size of the in situ adenocarcinoma, central hypoxic necrosis develops (**a**), which is the prerequisite for invasion (**b**)

As the primary tumor grows, usually the formation of new blood vessels cannot keep with this resulting in hypoxia. This is the time when tumor cells are faced with this problem and try to escape apoptosis induced by hypoxia. Some of these mechanisms have been elucidated. HIF1α is upregulated in areas of tumor hypoxia [24–28], and if translocated into the nucleus and bind to HIF1β can induce transcription of VEGF, thus increasing the formation of more blood vessels. Apoptosis is also inhibited by growth factors such as IGF and EGF, which are also induced by hypoxia [24, 29]. Carcinoma cells also escape apoptosis and cell death in hypoxic areas by reducing their metabolism and cell division [30]. In mouse models of lung adenocarcinomas driven by the mutated RAS oncogene, invasion was exclusively seen starting in areas of necrosis and hypoxia [31] (Fig. 3a, b). This fits wells with published data from human tumor research, showing that migration and epithelial to mesenchymal transition (EMT) are increased in hypoxic areas by the release of different proteins [32–36]. If each of these enzymes/proteins act in concert together or if each of these factors can act independently is presently unknown.

**Fig. 4:**
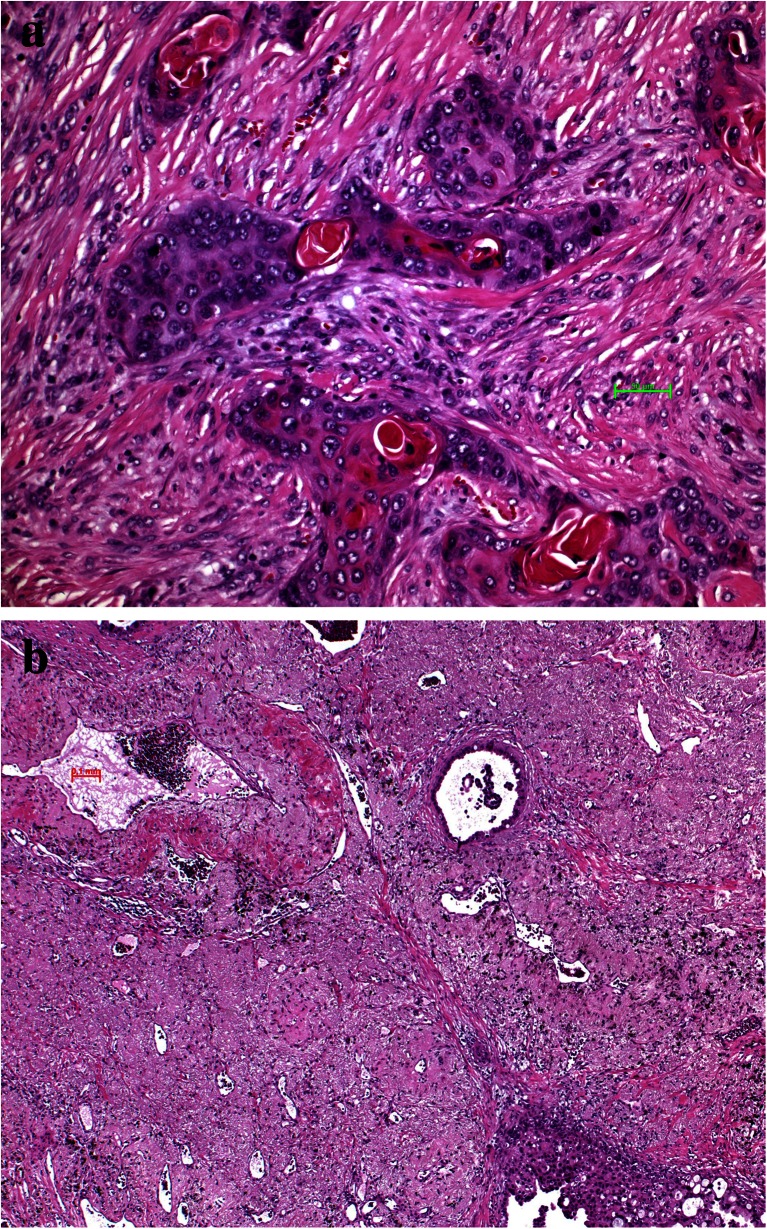
Desmoplastic stroma supports invasion and guides the carcinoma cells in this squamous cell carcinoma (**a**), whereas scar tissue inhibits invasion as in this adenocarcinoma example (**b**). The only way for the carcinoma cells is invasion into lymphatics, which happened in the center

**Fig. 5:**
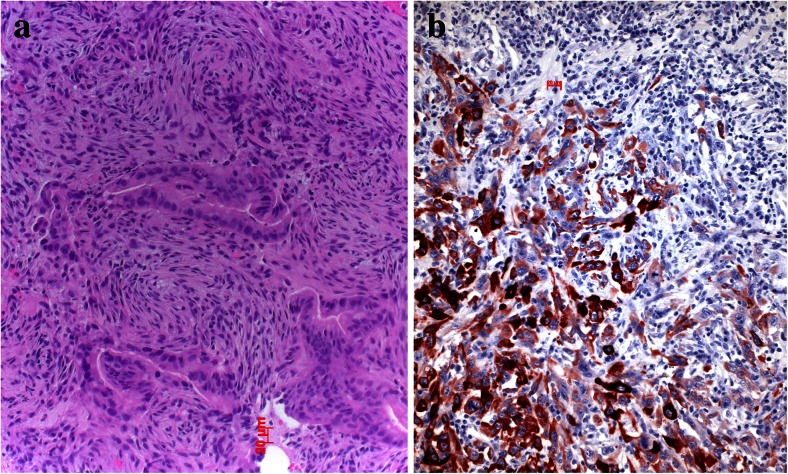
Epithelial to mesenchymal transition (EMT) is common in pleomorphic carcinomas of the lung (**a**); this can also be demonstrated by cytokeratin immunohistochemistry, showing epithelial tumor cells positively stained on the left side, whereas tumor cells at the right side have lost cytokeratin and acquired vimentin (**b**)

Macrophages also act together with fibroblasts and myofibroblasts to form either a stroma suitable for tumor cell invasion and migration or might inhibit migration (Fig. 2b). This can easily be evaluated by morphology. In case the stroma cells form a classical scar, this means inhibition for the tumor cells to migrate, whereas desmoplastic stroma is a form of stroma remodeling done by myofibroblasts, which enable tumor cells to migrate (Fig. 4a, b). Usually, fibroblasts in scars do not cooperate with tumor cells; myofibroblasts in contrary cooperate [37, 38]. In some cases, even tumor cells undergoing EMT directly form part of the desmoplastic stroma as in pleomorphic carcinomas (Fig. 5a, b) and occasionally also in SCLC [39]. Several studies have shown that tumor-associated “fibroblasts” (essentially myofibroblasts in the lung) are different from normal fibroblasts in the lung. They express different genes and proteins related to their function in cancer development. Specifically, MLH1 was upregulated, whereas COX1, FGFR4, Smad3, and p120 were downregulated [40]. Factors have been identified, which drive this differentiation of mesenchymal stem cells into myofibroblasts, namely, TGF-beta and IL-1β. TGF-β also induces the expression of α-SMA and FAP-α [41]. Serine protease fibroblast activation protein (FAP) promotes tumor growth in an endogenous mouse model of lung cancer driven by the K-rasG12D mutant. On the contrary, FAP depletion inhibits tumor cell proliferation indirectly by increasing collagen accumulation, decreasing myofibroblasts in number, and decreasing blood vessel density in tumors [42]. Most importantly, myofibroblasts also express metalloproteinases (MMPs) such as MMP-2, MMP-9, MMP-8, and MMP-7. So these cells actively take part in remodeling of matrix proteins. In addition, MMP-8 actively participates in the process of fibrocyte migration [43].

In this context, also changes of matrix proteins, their composition, and their orientation are important for tumor cell migration. Usually, the matrix is composed of several proteins such as different types of collagen (I, III, IV, and V; predominant collagen I), fibronectin, laminin, elastin, and osteonectin. These proteins provide stability to the stroma by their oriented deposition and network formation by cross-linking. They also serve as orientation molecules providing ligands for migrating leukocytes expressing adhesion molecules. This mechanism is also used by migrating tumor cells. As a further benefit, adhesion of SCLC cells to fibronectin, laminin, and collagen IV through β1 integrins confers resistance to apoptosis induced by standard chemotherapeutic agents. Adhesion to ECM proteins stimulated protein tyrosine kinase (PTK) activity in both untreated and etoposide-treated cells [44]. In non-small-cell lung carcinoma (NSCLC), osteonectin (SPARC; normally only in bronchial cartilages) is selectively synthesized by the cells of the tumor stroma in case of intratumoral hypoxia and acidity. Osteonectin proven by immunohistochemistry favors cancer cell invasion and migration [45].

Another important factor is the orientation and composition of matrix proteins. High amounts of elastin favor resistance for tumor cell migration, whereas high collagen and organized oriented deposition promote tumor cell migration. Conversely, direct cell contacts between tumor cells and fibroblasts can also create migratory-inhibitory matrix composed of unorganized collagens (I, III, IV, and V) and proteoglycans (biglycan, fibromodulin, perlecan, and versican). The thick desmoplastic fibers bundles can inhibit the migration and invasion of tumor cells [46]. Matrix protein deposition seems to be in addition regulated by a tumor suppressor gene, frequently lost in lung cancer, RBM5 (RNA-binding motif protein 5; chromosome 3p21.3). The encoded protein plays a role in the induction of cell cycle arrest and apoptosis. Loss of RBM5 causes upregulation of Rac1, β-catenin, collagen, and laminin, which in turn increase cell movement. Consequently, Rac1 and β-catenin correlate positively with lymph node metastasis in lung cancer patients [47]. Two other matrix proteins are less explored. Expression of periostin is associated with vimentin expression in the stroma or tumor epithelia and correlates with higher stage. The correlation of periostin expression with that of versican and collagen in advanced tumors was less obvious. Opposite to periostin, expression of elastin was associated with less advanced disease [39]. However, this observation still needs confirmation as well as more in-depth investigation for the function of these proteins.

So, invasion, tumor growth, and tumor cell migration of lung cancer cells are regulated by many different factors, as cytokines, adhesion molecules and receptors, and genes acting either directly on tumor cells or cells of the microenvironment.

### Escaping immune cell attack

Usually, tumor cells produce many modified proteins, which are recognized as foreign by dendritic cells and lymphocytes. Tumor cells are therefore attacked and destroyed by cytotoxic lymphocytes (CD8+). However, pulmonary carcinoma cells have developed different escape mechanisms to prevent this cytotoxic attack. By modulating the innate immune system, macrophages are preferentially forced to differentiate into M2 types as already explained. Another mechanism to protect tumor cell is to modify the pool of antigen presenting dendritic cells (DCs). Within dendritic cells, several functional quite opposite acting cell populations are discerned; conventional DC will present tumor antigens to T lymphocytes and force the production of cytotoxic T cells, whereas plasmocytoid and monocytoid DCs act as tumor protective cells. For example, Bombesin derived from SCLC inhibits IL-12 production by DC and their ability to activate T cells [48]. Tumor cells by secretion of TGF-β and prostaglandin E2 induce DCs to differentiate into regulatory DCs with a CD11c(low)CD11b(high) phenotype (also named plasmocytoid DC) and high expressions of IL-10, VEGF, and arginase I. These regulatory plasmocytoid DCs inhibited CD4+ T cell proliferation [49] and thus act protumorigenic. Another action to prevent cytotoxic lymphocyte attack is to induce an influx of regulatory T cells (Treg). Treg downregulate the production and influx of cytotoxic T cells and NK cells and promote immune tolerance [50–52]. Finally, also bone marrow-derived myeloid precursor cells (MDSCs) may downregulate a T cell-based immune reaction towards growing tumor cells by secreting arginase I [53]. Indoleamine 2,3-dioxygenase (IDO) and IL-6 seem to play a regulatory role for these MDSCs, as downregulation of IDO resulted in reduced lung tumor burden and improved survival in experimental settings. Loss of IDO resulted in an impairment of protumorigenic MDSC, whereas IL-6 recovered both MDSC suppressor function and metastasis susceptibility. In addition, vascular density was significantly reduced in Ido1-nullizygous mice [54].

In some carcinomas, preferentially in pulmonary squamous cell carcinomas, eosinophils are found in abundance. The role eosinophilic granulocytes play in NSCLC is not fully understood. It may be that variants of IL-17 (IL-17E) induce a helper 2 type of immune response, which in turn by the release of IL-4 and IL-5 causes tissue eosinophilia. In one study, it was shown that IL-17E has antitumor activity. Injections of recombinant IL-17E resulted in significant antitumor activity. Combining IL-17E with chemotherapy increased the antitumor efficacy in a xenograft model [55]. If eosinophils are directly acting cytotoxic against the tumor cells, for example, by releasing cytotoxic basic proteins was not explored in this study.

### Migration

**Fig. 6:**
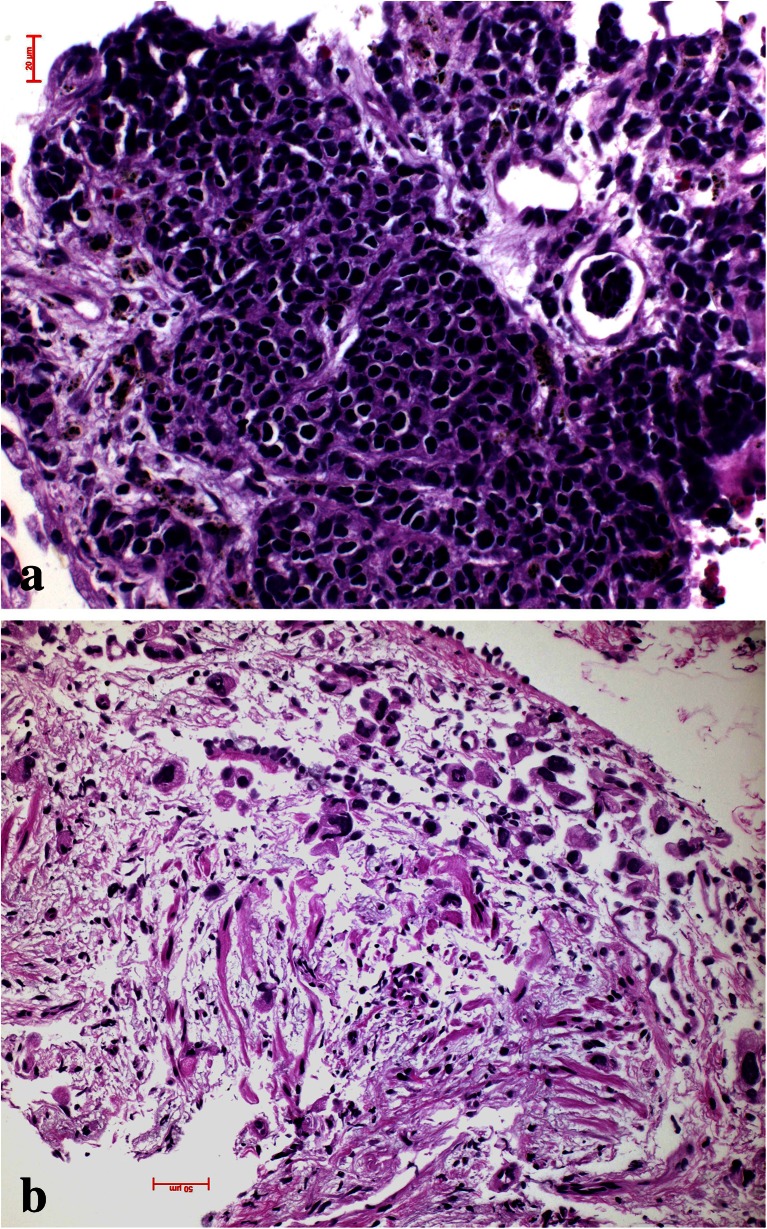
Tumor cell migration, **a** this small-cell neuroendocrine carcinoma moves in small-cell groups, whereas the adenocarcinoma (**b**) moves almost as single cell

**Fig. 7:**
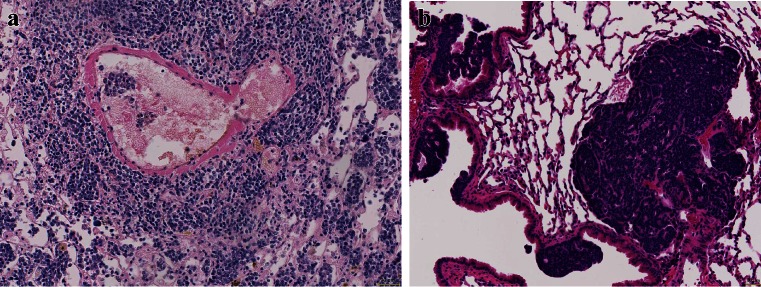
Tumor cell migration, **a** this mixed small- and large-cell neuroendocrine carcinoma migrates as single- or small-cell clusters, whereas the small-cell neuroendocrine carcinoma in **b** migrates in small complexes in this very early stage; experimental mouse model (slides provided by A. Gazdar)

**Fig. 8:**
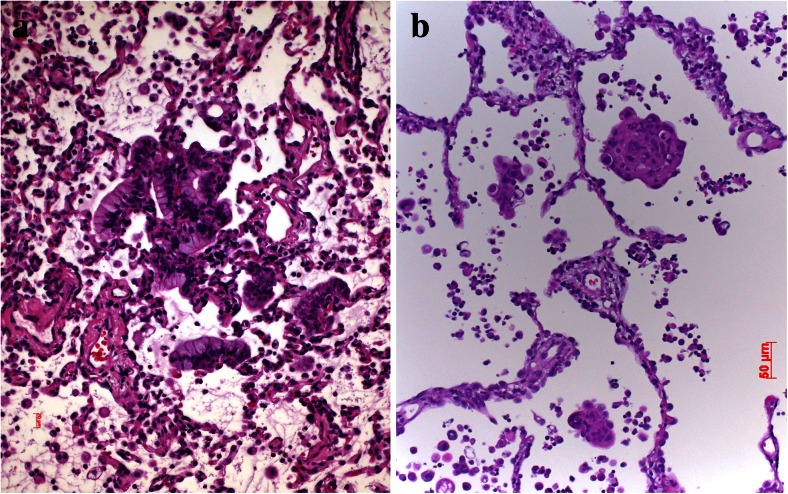
Tumor cell migration, **a** a mucinous adenocarcinoma moves in larger-cell complexes along the alveolar walls, still using the supply by the alveolar septa and **b** an unusual 3D complex of squamous cells moving as spheroids

**Fig. 9:**
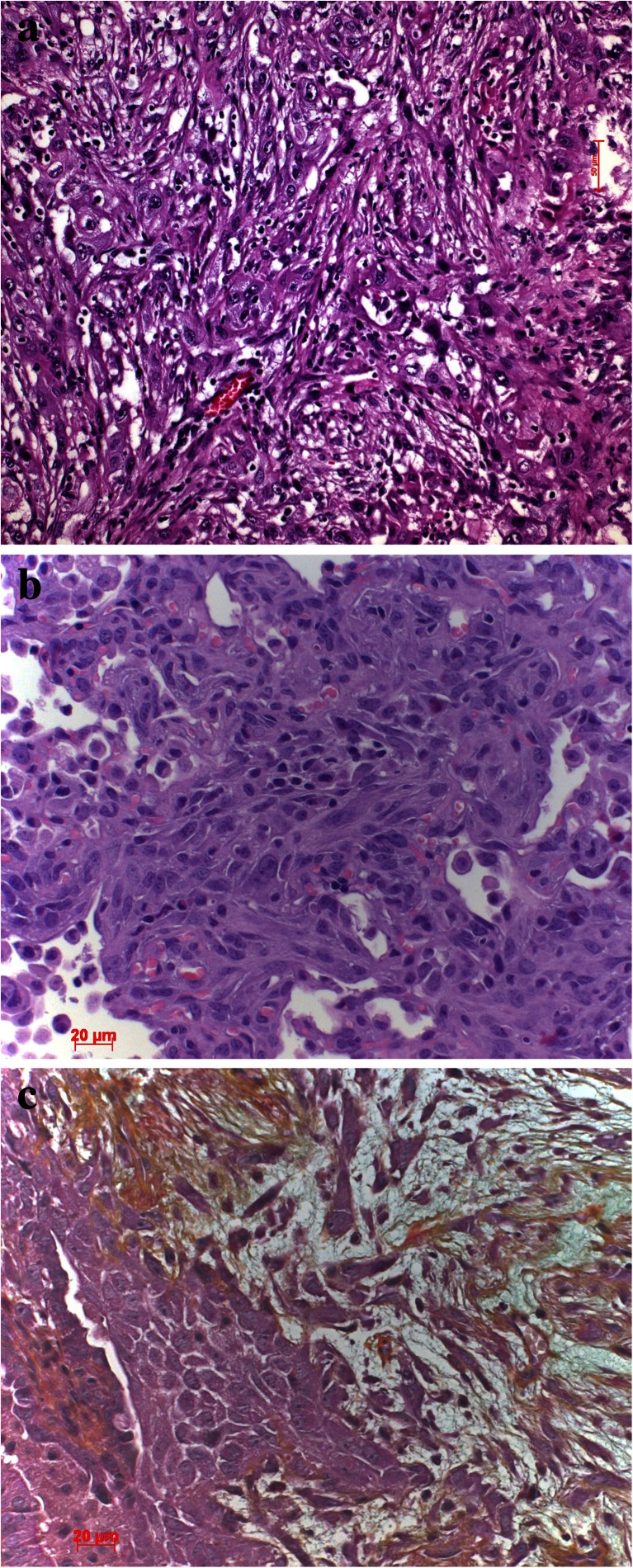
**a** EMT in a pleomorphic carcinoma with spindle cells and **b**, **c** EMT in mouse model of KRAS-induced adenocarcinomas with additional expression of mutant TP53. **c** Movat stain, which better demonstrates the invasion of the spindle tumor cells into the desmoplastic stroma

After having established the primary tumor and organized nutrition as well as protection for immune cell attacks, the tumor cells have to acquire changes to migrate to distant sites and establish metastasis. There are two different forms how tumor cells migrate, single-cell or small-cell cluster movement as it is seen in small-cell carcinoma as well as undifferentiated NSCLC and movement by large clusters of organized cells such as in acinar adenocarcinoma or some cases of squamous cell carcinoma (Figs. 6a, b, 7a, b, and 8a, b). For single cell and small clusters, migration seems to be much easier since single cells can more easily adapt, for example, a spindle cell morphology, which enables better movement. Tumor cells during migration reduce or even abolish cytokeratin filaments and increase/or *de novo* express α-actin and vimentin; this is commonly seen in pleomorphic carcinomas (Fig. 5b), carcinosarcomas, high-grade squamous cell and adenocarcinomas, and SCLC. Lung adenocarcinomas with high smooth muscle actin gene ACTA2 expression showed significantly enhanced distant metastasis and unfavorable prognosis. ACTA2 downregulation remarkably impaired *in vitro* migration, invasion, clonogenicity, and transendothelial penetration of adenocarcinoma cells without affecting proliferation. ACTA2 upregulation in lung adenocarcinoma cells was also connected to expression of c-MET and focal adhesion kinase (FAK), whereas ACTA2 targeting by small interfering RNAs (siRNAs) and short hairpin RNAs (shRNAs) resulted in loss of mesenchymal characteristics [56]. Migration within the stroma requires several changes in tumor cells, one is formation of invadopodia. Tyrosine kinase substrate 5 (Tks5) is a scaffolding protein necessary for the formation of invadopodia. There are different isoforms, some of them (short isoforms) associated with reduced other (long isoforms) increased metastasis [57–59]. Expression of Tks5 together with the expression of α-actin is further regulated by cortactin and neural Wiskott-Aldrich syndrome protein (N-WASP), which also regulate the expression of metalloprotease membrane type 1 matrix metalloprotease (MMP14) [58]. However, migration of tumor cells seems to be regulated by different genes, so probably, there is not a single mechanism for each tumor type, but more likely that tumor cells individually have adapted different mechanisms of migration protocols and used it during carcinogenesis. As an example, myosin heavy chain 9 (MYH9) and Copine III (CPNE3) positively correlate with the migration and invasion properties of lung cancer cell lines. If CPNE3 was knocked down, the metastatic abilities were inhibited in a mouse model. Also, CPNE3 protein expression levels were positively correlated with the clinical stage in NSCLC [60]. In another study, nestin protein expression significantly correlated with tumor size and lymph node metastasis in NSCLC and also poor survival in patients with adenocarcinoma. Nestin inhibition by shRNA decreased proliferation, migration, invasion, and sphere formation in adenocarcinoma cells [61]. One of the major studied mechanisms of tumor cell migration is EMT, which again is seen in tumors with single-cell or small-cluster migration type (Fig. 9a–c).

When looking up studies on EMT, a huge amount of published article can be found in databases. The major surprise is that different genes are associated with EMT. And even when focusing on lung cancer studies, there are still different genes found to trigger EMT. Some of the most often found EMT-associated genes are Twist, Snail, and TGFβ1.

Suppression of Twist expression in metastatic mammary carcinoma cells inhibits their ability to metastasize from the mammary gland to the lung. Ectopic expression of Twist resulted in loss of E-cadherin-mediated adhesion, activation of mesenchymal markers, and induction of cell motility, suggesting that Twist promotes EMT [62]. In another study, Twist was selectively associated with EGFR-mutated adenocarcinomas. Twist expressed in lung adenocarcinoma cell lines with EGFR mutation showed increased cell mobility. A decrease of EGFR pathway through EGF retrieval or inhibition of Twist expression by small RNA reversed the phenomenon. These findings supported that Twist promotes EMT in EGFR-mutated lung adenocarcinoma [63]. In the study by Pirozzi, the focus was on TGFβ1. They used two epithelial cell lines which acquired a fibroblast-like appearance when treated by TGFβ1. By inhibiting TGFβ1, vimentin and CD90 were downregulated and cytokeratin, E-cadherin, and CD326 were upregulated. TGFβ1 also upregulated Slug, Twist, and β-catenin, thus confirming EMT. Interestingly, also some stem cell markers as Oct4, Nanog, Sox2, and CD133 were overexpressed too, linking EMT to tumor stem cells [64]. Adhesion plays a major role in EMT; therefore, not surprisingly, studies have focused on Wnt, catenin, and GSK3β pathway. Loss of SARI (suppressor of AP-1, also called BATF2) expression initiates EMT, causing repression of E-cadherin and upregulation of vimentin in lung adenocarcinoma cell lines and in human lung adenocarcinomas. By knockdown endogenous SARI in a human lung xenograft-mouse model, multiple lymph node metastases developed. SARI has been shown to regulate EMT by modulating the (GSK)-3beta-beta-catenin signaling pathway [65]. In the study by Blaukovitsch, another pathway for EMT was shown; Snail and Twist were not involved in pulmonary sarcomatoid carcinomas but instead upregulation of c-Jun and consecutive overexpressions of Vimentin and Fascin were seen [66].

When dissecting sites of metastasis, the way EMT is regulated get more diverse; PREP1 accumulation was found in a large number of human brain metastases of various solid tumors, including NSCLC. PREP1 induces the expression of multiple activator protein 1 components including Fos-related antigen 1 (FRA-1). FRA-1 and PBX1 are required for EMT triggered by PREP1 in lung tumor cells [67]. The study by Shen showed that increased levels of long non-coding RNA metastasis-associated lung adenocarcinoma transcript 1 (MALAT1) promotes lung cancer brain metastasis by EMT, whereas silencing of MALAT1 inhibits lung cancer cell migration and metastasis in the brain [68]. So far, nothing similar was investigated for bone metastasis by pulmonary carcinomas.

**Fig. 10:**
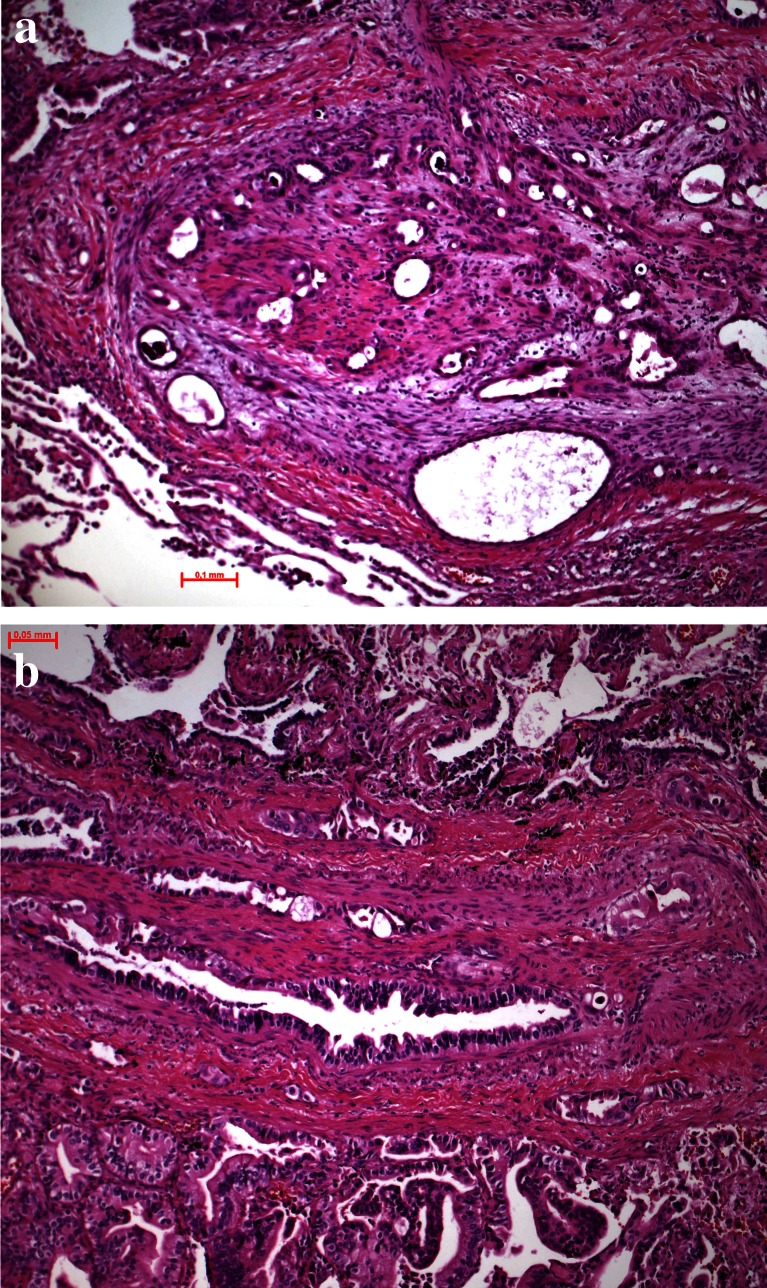
Vascular invasion, **a** tumor cells are scattered in acinar complexes within the intima of this pulmonary artery and **b** large acinar and papillary adenocarcinoma complexes can be seen within these blood vessels, demonstrating the other example of invasion as large tumor cell complexes

So far, we have focused on single-cell and small-cluster migration. However, in surgical pathology routine, most well-differentiated carcinomas including lung carcinomas move in large cell clusters; for example, acinar adenocarcinomas will show nice structured acini deep within the stroma and even within blood vessels (Fig. 10a, b). The mechanisms how these tumor cells manage their coordinated movement by retaining their epithelial structure is almost unknown. These carcinomas do not undergo EMT. Recently, in an investigation using drosophila border cells as a model, the processes of migration of large-cell complexes were elucidated. By RNAi silencing, 360 conserved signaling transduction genes were knocked down to identify essential pathways for border cell migration. The following four genes associated with TGF-beta signaling were identified: Rack1 (receptor of activated C kinase), brk (brinker), mad (mother against dpp), and sax (saxophone). Inhibition of Src activity by Rack1 may be important for border cell migration and cluster cohesion maintenance. Although this study focused on signaling pathways involved in collective migration during embryogenesis and organogenesis, these data could be the first step in understanding migration of carcinoma complex cancer metastasis [69].

## Vascular invasion, lymphatic/hematologic

### Blood vessels

Tumor cells orient themselves along adhesion molecules as expressed by matrix proteins, but in addition, they also sense for oxygen and most probably also orient themselves for higher oxygen tension [70]. Invasion into blood vessels is very similar to invasion into the stroma. Tumor cells have already learned to degrade proteins of the basal lamina at the epithelial border, and similar proteins form the matrix of small blood vessels. Forming holes into the basal lamina of these blood vessels is therefore easily done, and tumor cells migrate into the intima. However, a new problem arises for tumor cells within the circulation, shear stress due to tumor cell deformation in small blood vessels and the problem with coagulation.

Shear stress is usually well tolerated by those tumor cells which underwent EMT. Tumor cells expressing Vimentin and α-actin can adapt to the capillary diameters, but cells still expressing cytokeratins might burst. This is one of the reasons why a majority of tumor cells do not survive within the circulation [71]. With respect to coagulation, tumor cells on one hand have to avoid being trapped within a blood clot but on the other hand, will need a clot to slow down the speed of the blood stream, attach to the clot, and use it for extravasation [72]. Clot formation might be induced by tissue factor being produced and released by macrophages. Impairment of macrophage function decreased tumor cell survival without altering clot formation, demonstrating that the recruitment of functional macrophages was essential for tumor cell survival [73]. Another way how tumor cells might trigger clot formation has been demonstrated in mucinous adenocarcinomas. Mucins secreted by the tumor cells induced platelet aggregation and furthermore interacted with L-selectin and platelet-derived P-selectin without thrombin generation [74]. This interaction already points to the next step, adherence to vascular walls for extravasation. Coming back to tumor cell trapping by blood clots, it seems that carcinoma cells require the assistance of macrophages and granulocytes for fibrinolysis. In a study of lung carcinomas, fibrinolytic components as tissue plasminogen activators (t-PA) and the inhibitors PAI-1 and PAI-2 were all negative in tumor cells, whereas urokinase-specific antibodies stained loosely packed tumor cells and macrophages. Both PAI-1 and PAI-2 were most prominently expressed within interstitial and alveolar macrophages [75]. In another study analyzing pulmonary adenocarcinomas, a positive correlation was found between Ets-1 and urokinase-type plasminogen activator (u-PA) expression [76].

### Lymphatic vessels

Invasion into lymph vessels is easier than into blood vessels due to the tiny wall of the former. In addition, carcinoma cells might already enter the lymphatic stream by the interstitial channels of the lymph draining system. On the contrary, lymph vessels can easily be congested by tumor cells. This can reverse the lymph flow, which might explain unusual sites of lymph node metastasis and so-called skip lesions. In contrast to the situation within blood vessels, carcinoma cells in lymphatics have to deal with the immune system. So, survival is dependent on induction of immune cell escape mechanisms (see above).

Whereas carcinoma cells entering the blood stream might early onset distant metastasis and thus shorten overall survival of the patient [1], propagation of carcinoma cells along the lymphatics will set distant metastasis later. These tumor cells will set primarily metastasis within regional lymph nodes.

## Extravasation

Carcinoma cells have to escape the circulation. However, the process how tumor cells select their final destination is still not clear. A lot of information was gained from studies on homing mechanisms of lymphocytes and extravasation of granulocytes. The most important site are venules with high endothelia. First of all, the blood flow is reduced, which enables tumor cells to roll over the endothelia and express adhesion molecules. These adhesion molecules need to find their respective and specific receptors for adhesion. Once adhering to the endothelia, tumor cells have to activate the coagulation system for better and firm adherence, followed by production of holes between endothelia for migration out of the vessel lumen. Several factors have been identified, such as caveolin, which increases cell permeability. Loss of caveolin results in increased phosphorylation of VEGFR-2 and decreased association with the adherence junction protein, VE-cadherin. Loss of caveolin increases endothelial permeability and tumor growth [77]. Tumor cells might use different selectins such as E-selectin and P-selectin to adhere to specific sites on the endothelia of venules. Also, other selectins might be used, as has been shown by knockout of these selectins. PSGL-1, CD44, and CEA could be detected in SCLC cells. By intravital microscopy, SCLC cells were shown to roll along vessel walls mimicking leukocyte behavior [78].

## Creating the distant metastatic focus

It is well known that few tumor cells survive within the circulation. Even more, from those tumor cells, which survive and finally leave the circulation and settle at a distant site, only a small proportion progress and form metastatic nodules [71]. Usually, single-tumor cells die (probably with the exception of small-cell carcinoma cells), and small clusters form micronodules but do not grow further. Another enigma is the selection of metastatic sites. In general, lung cancer cells prefer the brain, bones, adrenal glands, and within lung carcinoma types, small-cell neuroendocrine carcinomas as well as adenocarcinomas metastasize into the brain, whereas squamous cell carcinomas prefer bones. What homing mechanisms are in action? And moreover, how carcinoma cells communicate with this new stroma? For example, in the brain, carcinoma cells need to organize their new homing by communicating with glial cells and also manipulate microglia to prevent attacks by immune cells and finally induce angiogenesis for their supply in nutrients and oxygen. In the following paragraphs, we will focus on different aspects of homing, extravasation, and creation of a metastatic niche in different organs.

To leave the circulation, lung cancer cells need signals, which seem to be specific for each organ. Some of these, such as E-selectin, are used in several carcinomas including breast and lung. Systemic inflammation may increase the expression of E-selectin, which mediate lung metastasis of an experimental breast cancer model [79]. Hyperpermeability is also a factor important for homing, because this slows down the blood flow and enable rolling of the tumor cells over the endothelia. Hyperpermeability is mediated by endothelial cell FAK, which upregulates E-selectin, leading to preferential homing of metastatic cancer cells to these foci [80]. Attachment of tumor cells however needs an activation of several other adhesion molecules. Once tumor cell attach on endothelia, they cause the induction of vascular cell adhesion molecule-1 (VCAM-1) and vascular adhesion protein-1 (VAP-1), which is dependent on tumor cell-clot formation, induced by tissue coagulation factors [81]. Also, changes in the cell to cell junctions of endothelia are necessary for the tumor cells, to move through interendothelial gaps. This is facilitated by an overexpression of angiopoietin-2 [82]. In addition, also MD-2, a coreceptor for toll-like receptor 4, triggers the formation of regions of hyperpermeability in mice by upregulating C-C chemokine receptor type 2 (CCR2) expression. The CCR2-CCL2 system induces the abundant secretion of permeability factors such as serum amyloid A3 and S100A8 [83].

Since all these investigations use different models and analyze different tumor tissues/or none, it is not surprising that other investigators found different acting molecules. Using cell cultures from an aggressive human squamous cell carcinoma, Chen subcultured different tumor clones and showed a different expression profile for members of the β1 integrin family. By the intravenous inoculation into SCID mice, the clonotypes differed in VLA-1 and VLA-2 expressions, where high levels of VLA-1 and VLA-2 display an increase in metastasis [84]. The group by Sadanandam identified 11 unique peptides specific for homing to lung, liver, bone marrow, or brain. Semaphorin 5A and its receptor plexin B3 were identified as relevant for homing to these organ sites [85]. A major factor for homing of carcinoma cells, including colon, lung, and breast, is the chemokine receptor CXCR4. The unique function of CXCR4 is to promote the homing of tumor cells to their microenvironment at the distant organ sites [86]. Acute inflammations seem to promote CXCR4 expression and may alter the lung microenvironment and prepare it for a metastatic “niche” [87]. CXCR4 inhibition reduced the influx of myeloid-derived cells and impaired lung metastases. CXCR4 is specifically expressed in stromal cells that prepare the protumor microenvironment [88].

Several other signaling proteins are also involved in metastatic homing and formation of a metastatic focus; however, how these different molecules interact with each other is not known.

In a study looking for the relationship of microRNA (miRNA) and metastasis, Liu et al. found that expression of miR-26a dramatically enhanced lung cancer cell migration and invasion. Matrix metallopeptidase 2 (MMP-2), VEGF, Twist, and β-catenin were upregulated. Phosphatase and tensin homolog (PTEN) was a direct target of miR-26a. They found that miR-26a increased AKT phosphorylation and nuclear factor kappa B (NFκB) activation. So, miR-26a enhanced lung cancer metastasis *via* activation of AKT pathway by PTEN suppression [89]. MALAT1-deficient cells are impaired in migration and form fewer tumor nodules in a mouse xenograft. Gene expression of MALAT1 is critical for lung cancer metastasis [90]. In addition, in another investigation, it was shown that MALAT1 cooperates with eIF4A1 and thymosin β4 in promoting metastasis in NSCLC [91].

### Angiogenesis

Angiogenesis at the metastatic site in one part follows the same principles as in the primary focus; however, there is one major problem. Whereas at the primary focus lung carcinoma cells cross-talk with stroma cells by mechanisms and transmitters which have been developed during the process of developing from the precursor lesion to *in situ* carcinoma to invasive carcinoma, this cross-talk is different in the new metastatic site. Brain glial cells or bone marrow stroma cells might response to other signals than those stroma cells within the lung. So, the major developmental step to establish a metastatic focus is communication with the stroma, and further, more communication might be different depending on the location. In one investigation, a bridge was built between angiogenesis at the primary and metastatic sites. CXCL12 was expressed in tumor cells and in tumor vessels; CXCR7 was expressed by tumor and endothelial cells in the primary tumor and in the brain metastasis. CXCR4 showed a nuclear positivity in all samples, but only CXCL12 expression in tumor endothelial cells was significantly correlated with shorter survival [92].

In interaction with stroma, there are no published data which could highlight general mechanisms by which lung carcinoma cells communicate with their stromal counterparts; however, communication at different organ sites have been studied and therefore will be discussed in the following paragraph.

### Metastasis

When discussing metastasis, many questions arise, which are still incompletely answered. When does metastasis occur? Is there a need for a certain size of the primary tumor that cells leave and start migrating? Are tumor cells randomly moving out from the tumor or are these selective clones, and are these genetically different from the dominant clone? Do carcinoma cells move collectively or as single cells? These questions have been discussed extensively in the literature, but especially when comparing metastasis in lung cancer, several good examples are there to answer at least some of these questions.

**Fig. 11:**
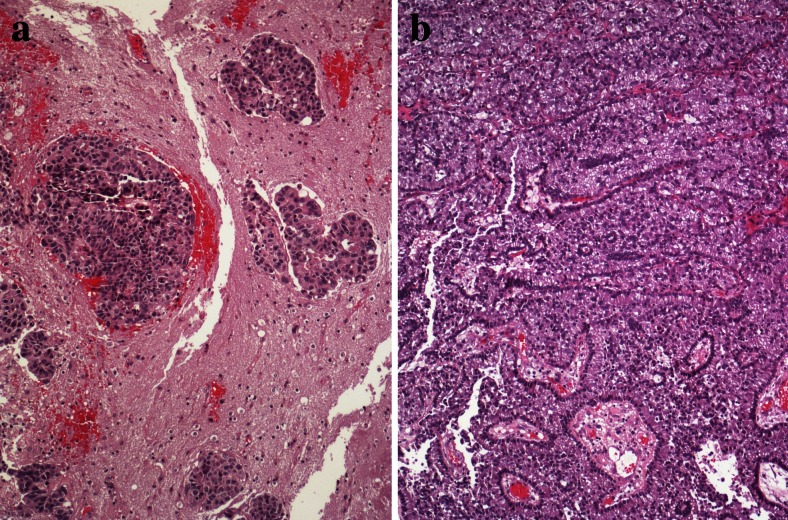
Brain metastasis, **a** cells of an adenocarcinoma interacting with astrocytes and microglial cells and **b** large adenocarcinoma complexes have acquired huge areas of the brain but in addition, imitate ependymal structures

Small-cell neuroendocrine carcinoma has some unique features. When looking at the invasion front, it is evident that this carcinoma prefers migration of single-cell and small-cell clusters composed of three to five cells (Figs. 6a, and 7a). In blood and lymphatic vessels, SCLC usually presents with single cells or small clusters of cells. Quite common is the finding of several large brain metastases and a very small primary tumor, which might even escape the detection by HRCT. This raises the question of early migration of carcinoma cells from the initial focus and setting metastasis early in the tumor development. In contrast, squamous cell carcinoma can form a large primary tumor, and when surgically removed has not formed metastasis; even some cases have not set regional lymph node metastasis. Migrating squamous cell carcinoma (SCC) often form large complexes of cells and when seen intravascular again present with large cell complexes. So, both extremes do occur in lung carcinomas. In adenocarcinomas, both types of migration and metastasis do occur, usually large migrating complexes of well-differentiated acinar or papillary adenocarcinomas (Fig. 11a), and small-cell clusters of solid or mucinous adenocarcinomas. The aspect of genetic heterogeneity in primary and metastatic tumor clones has been investigated in studies comparing primary and metastatic carcinomas. More importantly, it seems that not only primary carcinomas are different from metastasis but also metastases are different among each other. To be clear, driver mutations or general genetic aberrations in primary and secondary tumors are still identical, but additional genetic modifications arose within the metastases.

When looking up the frequency of metastasis of lung carcinomas, there are some preferential sites, as bone 34.3 %, lung 32.1 %, brain 28.4 %, adrenals 16.7 %, and liver 13.4 % [3]. We will discuss some of these, depending on the availability of research data.

#### Brain metastasis

Research coming from brain metastasis of breast and NSCLC have raised several important findings. First metastatic carcinomas can colonize the brain in different ways. Renal cell carcinomas most often form metastases which are well circumscribed and grow not out of the microglia pseudocapsule, whereas SCLCs tend to form small metastatic foci and tumor cells grown into the microglia pseudocapsule and beyond into brain parenchyma. This has nicely been demonstrated by a coculture system consisting of an organotypic mouse brain slice and epithelial cells embedded in matrigel (3D cell sphere) [93]. In addition by the same group of researchers, it has been shown that microglia support invasion and colonization of brain tissue by breast and lung cancer cells. This is under the control of the Wnt pathway, as upregulation of Dickkopf-2, an inhibitor of Wnt, inactivates the prometastatic function of microglia. Similar to tumor dentritic cell interaction, bacterial lipopolysacharide shifts tumor-educated microglia into a classical M1 phenotype, reduces their proinvasive function, and unmasks inflammatory and Wnt signaling as the most strongly regulated pathways [94]. Several factors have been identified as being specifically involved in regulating brain metastasis, but so far, these are still isolated factors, and the main question how these different factors interact remains unanswered.

Among the different cells of the brain, astrocytes seem to serve invading carcinoma cells. Astrocytes secrete matrix metalloprotease-2 (MMP-2) and MMP-9 that proactively induced human lung and breast tumor cell invasion and metastasis formation [95]. In addition, factors from the coagulation cascade are important. Plasmin acts as a defense against metastatic invasion by converting membrane-bound astrocytic FasL into a paracrine death signal for cancer cells and by inactivating the axon path-finding molecule L1CAM, which metastatic cells express for spreading along brain capillaries and for metastatic outgrowth. But, metastatic carcinoma cells from lung and breast secrete neuroserpin and serpin B2 to prevent plasmin generation and its metastasis-suppressive effects [96]. Within the Wnt pathway, LEF1/TCF4 acts independently of β-catenin in cerebrally metastasized human lung adenocarcinomas [97]. Downregulation of E-cadherin was also observed in a majority of adenocarcinoma and small-cell lung cancer samples. LOH of the CDH1 gene was frequently found in SCLC. Altered expression of Dishevelled-1, Dishevelled-3, E-cadherin, and beta-catenin were present in brain metastases of SCLC and adenocarcinoma, again pointing to the importance of the Wnt signaling [98]. In another study, peritumoral brain edema was shown to be associated with increased β-catenin, E-cadherin, and decreased CD44v6 and caspase-9 expressions in brain metastatic squamous cell carcinoma [99]. These findings were confirmed in another study showing a significant correlation of increased collagen XVII in adenocarcinoma and increased caspase-9, CD44v6, and decreased cellular apoptosis susceptibility protein (CAS) and Ki-67 in squamous cell carcinoma in brain metastasis [100].

Interestingly, when looking up adenocarcinomas with ALK1 rearrangement, FGFR1 gene amplification correlated significantly with brain metastases. Although in these cases there were also higher numbers of visceral metastases, FGFR1 amplifications in brain metastases of adenocarcinomas were fivefold more frequent than in the primary tumors [68]. Also, a cross-talk of EGFR-MET was reported in adenocarcinomas with brain metastasis. This was not a direct interaction but a signaling *via* the activation of mitogen-activated protein kinases (MAPKs). EGFR-MET cross-talk was independent from the mutation status of EGFR. MET signaling promoted migration and invasion. MET inhibition decreased the incidence of brain metastasis [101]. Also, CXCR4 seems to play a role in brain metastasis. CXCR4 protein was highly overexpressed in patients with brain-specific metastasis but significantly less in NSCLC patients with other organ metastases and without metastases [102]. Another factor ADAM9 levels were relatively higher in brain metastases than the levels observed in primary lung tumors. ADAM9 regulates lung cancer metastasis to the brain by facilitating the tPA-mediated cleavage of CDCP1 [103]. In a subsequent study, it was shown that ADAM9 regulated miR-218, which targets CDH2 in aggressive lung cancer cells. The downregulation of ADAM9 upregulated SLIT2 and miR-218, which together downregulated CDH2 expression. This study revealed that ADAM9 activates CDH2 through the release of miR-218 inhibition on CDH2 in lung adenocarcinoma [104].

A lot of interesting studies focused on the comparison of genomic alterations between primary lung carcinomas versus brain and bone metastases. The hypothesis is that there might be a clonal diversity between these two. It is still not clear if genetic differences between the primary tumor and the metastatic site is a primary event; i.e., clones are existing within the primary tumor, or if these are secondary events, reflecting the interaction of the carcinoma cells with the microenvironment and the cells therein. Wrage et al. found gene copy number variations between primary and CNS metastasis by array CGH. Genes with amplified copy numbers in primary and metastatic tumors were related to DNA replication and mismatch repair. Genes only amplified in the metastatic tumor were related to leukocyte migration and organ development. Genes with a lower copy number in the metastatic tumor were related to proteolysis, negative regulation of cell proliferation, and cell adhesion [105].

As with many marker studies focusing on single gene/protein, a selection bias or just an overinterpretation does occur. We already discussed MALAT1 in the setting of invasion and metastasis. Shen and coworkers found higher levels of MALAT1 in brain metastases compared to other extrapulmonary sites. In the in vitro experiments, it turned out that the major function of this lnRNA is EMT [68]. What can be concluded is that EMT seems to be required for tumor cells invading brain tissues, but MALAT1 is not a brain metastasis gene per se. A similar investigation searched for brain metastasis genes and came up with an EMT regulator; pre-B cell leukemia homeobox (Pbx)-regulating protein-1 (Prep1) overexpression triggered EMT, whereas PREP1 downregulation inhibits the induction of EMT in response to TGF-β. PREP1 modulates the sensitivity to SMAD3 and induces the expression of Fos-related antigen 1 (FRA-1). Both FRA-1 and PBX1 are required for the mesenchymal changes triggered by PREP1 in lung tumor cells. PREP1-induced mesenchymal transformation correlates with increased lung colonization, and PREP1 accumulation was found in human brain metastases [67]. Similarly, migration seems to play a role in brain metastasis, not surprisingly, since migration is often associated with EMT. Han et al. showed that knockdown of KDM5B and SIRT1 genes specifically inhibits lung cancer cell migration in vitro. SIRT1 was highly expressed in brain metastasis. Using other lung cancer cell lines, the authors showed that the function of SIRT1 correlates with cell migration [106]. There are not many studies looking for the influence of molecules of the adhesin family. Nasser and colleagues investigated E-cadherin expression. Low E-cadherin expression was associated with increased risk of developing brain metastasis. By treating tumor in a mouse model with pioglitazone, a peroxisome proliferator-activated receptor γ-activating drug prevented loss of E-cadherin expression and reduced expressions of MMP9 and fibronectin and furthermore, the development of brain metastasis [107].

Two studies showed an association of genotypic variants with brain metastasis. In the study by Li et al. [108], genotypes for AKT1 and PI3K were associated with brain metastasis risk (AKT1 rs2498804, AKT1 rs2494732, and PIK3CA rs2699887). In another study by Li [109], genotype variations for SMAD6 (rs12913975) and INHBC (rs4760259) were associated with risk of brain metastasis.

Whereas most studies on brain metastasis focused on the most common lung adenocarcinoma, Paik and coworkers studied squamous cell carcinomas, which rarely present with brain metastasis. They found “truncal” PTEN loss and PI3K-aberrant tumors to be associated with brain metastases. There was also a genetic heterogeneity between lung primaries and brain metastases [110].

#### Lung metastasis

Although pulmonary metastasis is common in adenocarcinomas as well as SCLC, not much is known about specific molecular mechanisms. In the study by Ruoslathi, connexin-43 was identified as adhesion molecule facilitating “homing” to the lung endothelial cells. Connexin-43 was highly upregulated in tumor cells during endothelial cell contact [111].

#### Bone metastasis

In bone metastasis, research reports focused on two different aspects of colonization, homing mechanisms and interaction of carcinoma cells with the bone/bone marrow stroma. In the work of Yang, PDGFRβ was found to be the main tyrosine kinase expressed in BM stromal ST-2 and MC3T3-E1 preosteoblastic cells. Incubation of ST-2 and human BM endothelial cells with sunitinib, a PDGFRβ inhibitor, led to growth inhibition and induction of apoptosis. Sunitinib produced extensive disruption of tissue architecture and vessel leakage in the BM cavity. Pretreatment of ST-2 cells with sunitinib hindered adhesion to lung cancer cell lines. Pretreatment of mice with sunitinib before intracardiac inoculation of A549M1 or H460M5 cells caused marked inhibition of tumor cells homing to bone, whereas no effect was found when tumor cells were pretreated before inoculation [112]. Several studies focused on the reaction of osteoclasts, which cell type seems to be important for creating a metastatic “niche” for tumor cells in the bone.

Knockdown of DDR1 by siRNA showed reduced invasiveness in collagen matrices and increased apoptosis. Conditioned media of DDR1 knockdown cells decreased osteoclastogenic activity *in vitro*. In a bone metastasis model lacking DDR1, decreased metastatic activity and reduced tumor burden and osteolytic lesions were achieved. These resulted also in a substantial reduction of tumor cells reaching the bone compartment [113]. Vincent et al. showed induction of TGF-β-dependent osteoclastogenic bone resorption and enhanced stroma-dependent metalloproteolytic activities by TCF4 and PRKD3 and anchorage-related proteins MCAM and SUSD5 resulting in aggressive osseous colonization [114]. In another study, stromal cell-derived factor-1 (SDF-1) secreted by osteoblasts and bone marrow stromal cells enhanced the invasiveness of lung cancer cells by increasing MMP-9 expression through the CXCR4/ERK/NFκB signal transduction pathway [115]. In another approach, researchers focused on miRNAs associated with bone metastasis of lung cancer. Seven miRNAs were downregulated and 21 miRNAs were upregulated in lung adenocarcinoma. Functional bioinformatic annotation analysis indicated that the MAPK, Wnt, and NFκB signaling pathways, as well as pathways involving the matrix metalloproteinase, cytoskeletal protein, and angiogenesis factors, are involved in orchestrating bone metastasis [116]. Finally, in the study by Luis-Ravelo, the function of RHOB, a small GTPase, was investigated. Gene silencing of RHOB prevented metastatic activity in a systemic murine model of bone metastasis. Consistently, high RHOB levels promote metastasis progression [117].

**Fig. 12:**
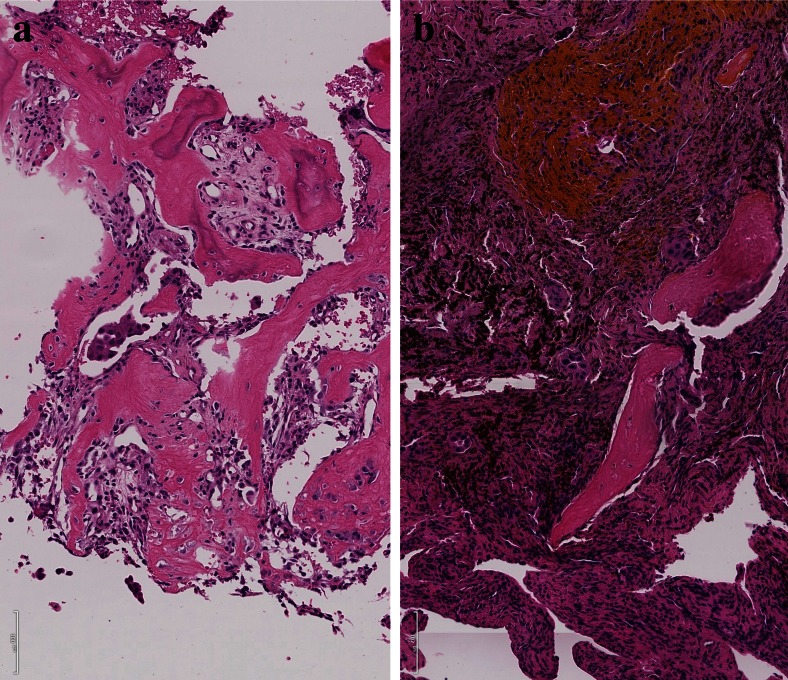
Bone metastasis, **a** adenocarcinoma cell complexes have induced an impressive activity of osteoclasts, resulting in lytic bone lesions; **b** adenocarcinoma metastases have induced bleeding and a massive inflammatory reaction, which also results in lytic bone lesions

A new promising aspect came with the demonstration that the RANK-RANK ligand system regulates the activity of osteoclasts. CCL22 upregulated receptor activator of nuclear factor-κB ligand (RANKL) in osteoclast-like cells which subsequently induced cell migration and also enhanced phosphorylation of protein kinase B/Akt and extracellular signal-regulated kinase (ERK). This suggests that osteoclasts may promote bone metastasis of cancer cells expressing CCR4 in the bone marrow by producing its ligand CCL22 [118]. Lung cancer metastases to bone produce primarily mixed osteolytic/osteoblastic lesions (Fig. 12a, b). Treatment with RANK antibody limited the formation of lytic lesions and inhibited the rate of in vivo tumor growth [119]. The study by Kuo and coworkers studied the regulation and interaction of parathyroid hormone-related protein (PTHrP). The authors showed that miR-33a levels are inversely correlated with PTHrP expression. The reintroduction of miR-33a reduces the production of osteoclastogenesis activator RANKL and macrophage colony-stimulating factor (M-CSF) on osteoblasts, while the expression of PTHrP was decreased. In addition, miR-33a-mediated PTHrP downregulation results in decreased IL-8 secretion and contributes to decreased lung cancer-mediated osteoclast differentiation and bone resorption in experimental setting [120]. Peng and colleagues showed upregulated RANKL, RANK, and OPG in NSCLC cell lines and in tumor tissues with bone metastasis. Migration and invasion were significantly enhanced by recombinant human RANKL and transfection of RANKL cDNA and were impaired after OPG was added. Differential expressions of RANKL, RANK, and OPG were shown to be associated with the metastatic potential of human NSCLC to skeleton [121]. This was confirmed by the study of Miller. Tumor cell-mediated osteolysis occurs through induction of RANKL. The authors tested this hypothesis in novel NSCLC bone metastasis mouse models. They found that OPG-Fc reduced the development and progression of osteolytic lesions. OPG-Fc plus docetaxel in combination resulted in significantly greater inhibition of skeletal tumor growth compared to either single agent alone. The inhibition of RANKL reduced osteolytic bone destruction and skeletal tumor burden [122].

Dougall and coworkers used denosumab, a fully human monoclonal antibody against RANKL, and demonstrated prevention or delay of skeletal-related events in patients with solid tumors that have metastasized to bone. Besides the role of RANKL in tumor-induced osteolysis, bone destruction, and skeletal tumor progression, the authors also provided arguments for a direct pro-metastatic effect of RANKL, as RANKL also stimulates metastasis via activity on RANK-expressing cancer cells, resulting in increased invasion and migration [123].

#### Pleural metastasis

Lung carcinomas frequently metastasize to the pleura. Especially, adenocarcinomas because of their peripheral location invade early on the pleura. Interestingly, when comparing adenocarcinomas with known driver mutation, it is evident that adenocarcinomas with EML4-ALK1 rearrangement have a higher propensity for pleura metastasis and malignant effusion [124]. In one study, it was proposed that the 216G/T polymorphism of the EGF receptor may play a role in pleural metastasis by overexpressing the protein [125].

### Lymph nodes

Although lymph nodes are among the first metastatic foci of lung carcinomas, much less is known about specific molecular events in facilitating this colonization. Peng et al. studied the effect of hypoxia and chemokines. CCR7 expression correlated positively with HIF-1α and HIF-2α, and all together correlated with lymph node metastasis. It was shown that hypoxia induced HIF-1α and HIF-2α expression, which upregulated CCR7; inhibiting HIF-1α or HIF-2α resulted in decreased CCR7 expression and furthermore in inhibition of tumor cell migration and invasion [126]. However, it seems obvious that more than these three molecules are involved, which has been shown by a study on genetic aberrations. Gains at 7q36, 8p12, 10q22, and 12p12; loss at 4p14; and the homozygous deletions at 4q occurred significantly more frequent in SCC from patients with lymph node metastases only. Gains at 7q, 8p, and 10q were restricted to SCC with lymph node metastasis, and gain at 8q was restricted to patients with distant metastasis [127].

In summarizing our knowledge in the metastatic process in lung carcinomas, it can be stated that many mechanisms and involved genes/proteins have been identified, but the major breakthrough is still not achieved. The major problem is that the process of metastasis has so many steps that we still do not overlook the interactions of hypoxia, migration, EMT and MET, homing, interaction with stoma cells, and preparation of the metastatic niche, which probably occur not as a time sequence but more likely in parallel.

Finally, we will not discuss metastasis to the lung, although there are complementing factors hidden, which might help in understanding homing mechanisms, such as the exclusive metastasis of glioblastomas and meningosarcomas to the lung. But, this would too much expand this review.
